# Exploring the Link Between Emotional Child Abuse and Anorexia Nervosa: A Psychopathological Correlation

**DOI:** 10.7759/cureus.5318

**Published:** 2019-08-04

**Authors:** Tehrima Rai, Pranita Mainali, Ali Raza, Junaid Rashid, Ian Rutkofsky

**Affiliations:** 1 Pediatrics, California Institute of Behavioral Neurosciences and Psychology, Fairfield, USA; 2 Psychiatry, Washington DC VA Medical Center, Washington DC, USA; 3 Internal Medicine, California Institute of Behavioral Neurosciences and Psychology, Fairfield, USA; 4 Medicine and Histopathology, California Instititute of Behavioral Neurosciences and Psychology, Fairfield, USA; 5 Psychiatry, California Institute of Behavioral Neurosciences and Psychology, Fairfield, USA

**Keywords:** child abuse, anoraexia nervosa, adult survivors of child abuse, psychopathology, feeding and eating disorders

## Abstract

Eating disorders (ED) are well known psychiatric disorders associated with dysregulated eating behaviors and related thoughts and emotions. Common eating disorders are bulimia nervosa (BN), anorexia nervosa (AN), and binge eating disorders (BED). There is an active link between child abuse and eating disorders, emotional child abuse being the important subtype of CA and has a strong comorbid psychopathological relationship with EDs, including AN. The PubMed database was searched for the related articles about child abuse, including emotional childhood maltreatment and their psychopathology associated with EDs, especially AN. No filters were used for the date of publication and article types. Childhood abuse, including physical, sexual, and emotional maltreatment, has an active link with psychopathology associated with dysregulated eating behaviors. However, emotional childhood maltreatment including emotional abuse, neglect, and/or exposure to intimate partner violence (IPV) has been least studied, but studies have shown a strong relationship with the symptoms of anorexia nervosa such as weight concern, negative self-image, and maladaptive emotional response. Emotional dysregulation is the crucial psychopathological factor involved in mediating the effects of emotional childhood maltreatment and symptoms of anorexia nervosa and is strongly associated with long-term morbidity in patients with AN. Conducting more clinical studies in the future would help explore the temporal causation, and this association may help the practitioners to develop new diagnostic and therapeutic strategies in the management of AN.

## Introduction and background

Eating disorders (EDs) are serious and debilitating psychiatric disorders and cover a broad range of subtypes including anorexia nervosa, binge eating disorders, bulimia nervosa, and other specified eating and feeding disorders (previously known as not otherwise specified eating and feeding disorders). These EDs tend to have onset in childhood or adolescent life, and those suffering from these disordered behaviors experience long-lasting morbidity associated with it, including recurrent hospital admissions. The hospitalization rate due to EDs and the length of hospital stay has increased by 40% or greater for children and adolescents in Canada and the US since the early 2000s [1]. Lifetime prevalence estimates of DSM-IV anorexia nervosa, bulimia nervosa, and binge eating disorder are 0.9%, 1.5%, and 3.5% among women and 0.3% 0.5%, and 2.0% among men, respectively [2]. EDs are associated with psychopathological impairment and disability and are often undertreated. In this regard, eating and feeding disorders should have a public health concern, and all underlying factors influencing these disorders must be explored.

Child abuse, which includes sexual, physical and emotional abuse, child physical and emotional neglect, and child maltreatment, is a serious social problem globally. The BECAN study showed lifetime exposure rate of maximum 83.2% for psychological violence, 76.3% for physical violence, 18.6% for sexual violence, and 9.8% for sexual contact violence; and lifetime prevalence for self-reported neglect was maximum of 20.3 % [3]. Child abuse and neglect can cause dysregulation in behaviors both externally and internally leading to serious psychological health issues including mood and anxiety disorders, personality disorders, and alcohol use disorders, all of which have been found to occur concurrently at high rates among adolescents and adults with eating and weight-related pathology [4-5]. Among all types of child abuse, emotional abuse (EA) and emotional neglect (EN) are distinct in physiological and psychological impacts [6]. Also, emotional child abuse and neglect, and exposure to intimate partner violence (IPV) are challenging to recognize and often underreported.

Anorexia nervosa (AN) is one of the eating disorders, which has been linked with a history of child abuse, child neglect, and child maltreatment. Interest in the role of emotion regulation difficulties in the development, maintenance, and treatment of individuals with anorexia nervosa (AN) is burgeoning [7]. Theoretical models explaining the problems in emotional regulation in those suffering from AN specify the role of all influential factors that can lead to emotional dysregulation either by precipitating it or predisposing to it [8-9]. There have been mixed results explaining the correlation between various types of child abuse and child neglect with anorexia nervosa especially the linkage of emotional abuse and neglect with anorexia nervosa has been least transparent in previous studies. No published literature reports explicitly on whether those with and without a history of AN differ on other types of childhood adversity including emotional and physical neglect and abuse. The exclusion of these different forms of maltreatment has been noted as a weakness in the literature [10]. Exploring the link between emotional dysregulation and anorexia nervosa can help health practitioners emphasize on treatment and management strategies that can address child abuse and its impact on emotional disordered behaviors leading to eating behaviors in patients with AN and decrease long-term morbidity.

## Review

Methods and results

PubMed database was systemically searched for related articles about child abuse and its psychopathological association with EDs, with particular emphasis on AN. Mesh keywords used were child abuse, psychopathology, and adult survivors of child abuse, feeding and eating disorders, and anorexia nervosa. After searching without any restriction of geographical distribution and dates of publications, 28,846 articles were found for Mesh keyword ‘child abuse’, 28,777 articles for ‘feeding and eating disorders’, 12,436 articles for ‘anorexia nervosa’, and 1911 articles for ‘adult survivors of child abuse’. The search team found 277 articles for combined Mesh keywords ‘child abuse’ and ‘feeding and eating disorders’, 98 articles for combined mesh keywords ‘child abuse’ and ‘anorexia nervosa’, 38 articles for combined mesh keywords ‘adult survivors of child abuse’ and ‘feeding and eating disorders’, 138 articles for combined mesh keywords ‘child abuse’ and ‘psychopathology’, and seven articles for combined mesh keywords for ‘adult survivors of child abuse’ and ‘anorexia nervosa’. Moreover, duplicate and irrelevant articles appeared in the search were excluded. In this review, all types of studies, including clinical trials, review article, and case reports were included without limiting the study to one specific category.

Psychopathology of Childhood Maltreatment

Childhood trauma is known to be associated with several psychiatric disorders. It leads to negative memory bias, which is strongly related to psychiatric co-morbid conditions. Individuals who experienced childhood abuse and/or neglect have problems in the emotional thought process and disturbed behaviors in response to stressful life conditions. Interestingly, early life traumatic events lead to increased mood and anxiety disorders, and one of the essential underlying pathologies is hyperactivity of the hypothalamic-pituitary axis (HPA). Also, the victims of childhood maltreatment, abuse and/or neglect exhibit attenuated cortisol awakening response in stressful life conditions and have the low meaning of life leading to depressive symptoms and disordered emotional behaviors and eating attitudes. Additionally, individuals with single nucleotide polymorphism (SNPs) in specific genes are more susceptible to adverse psychopathological effects of child abuse and maltreatment as compared to those not having SNPs.

Child Abuse and ED Severity

Child abuse is one of the most influential risk factors in increasing the severity of EDs. Individuals with EDs have multiple characteristics like pre-occupation with shape and weight and maladaptive eating practices, and severity of these symptoms are directly related to all types of child abuse and neglect. Individuals who endured severe kind of victimization feel overwhelmed in stressful environments and use maladaptive coping mechanism, including disordered eating behaviors. In short, there is a direct dose-response kind of link between childhood maltreatment and ED symptom severity, leading to increased long-term morbidity and frequent hospital admissions in later adulthood.

Emotional Dysregulation as a Mediating Factor in AN

Patients with AN have higher levels of co-morbid psychopathological conditions like depression, anxiety disorders, obsessive-compulsive disorders, and sensitivity in interpersonal relationships; and all are linked by disturbed emotional processing. They have a high prevalence of emotional neglect and abuse as compared to the general population. This linkage is mediated by emotional dysregulation, which is a significant variable in the development of disordered eating behaviors in AN. These behaviors tend to evolve to inhibit the emotions of anger and disgust as a part of maladaptive attitudes. Among the subtypes of AN, emotional neglect is strongly associated with binge-purge type AN as compared to restrictive type AN.

CBT Role in Targeting Emotional Dysregulated Behaviors in Patients with AN

As the learning about related psychopathology of child abuse and EDs is becoming clear, it calls for the need to address childhood maltreatment in the evaluation, treatment, and management options of AN. For evaluation, measuring scales like Invalidating Childhood Environments Scale (ICES) can be used to assess the invalidating parental behaviors and its impact on stress tolerance level, the relationship between childhood experience and eating pathology. Also measuring scales for self-esteem, anorectic psychopathology, depression, anxiety, and social support levels should be used in routine clinical practice for a comprehensive assessment. This way, it can help physicians screen high-risk patients and define behavioral therapies to facilitate emotional recovery via behavioral therapies; hence decreasing long term morbidity and frequency of hospitalization in patients with AN.

Discussion

Psychopathology of Child Abuse

**Figure 1:**
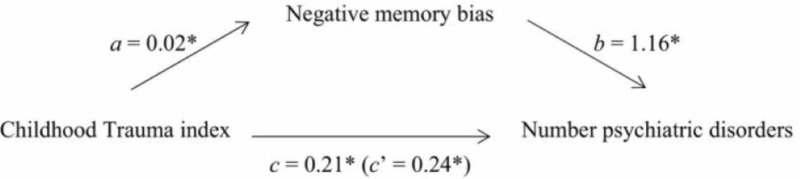
Impact of child abuse on memory leading to psychiatric disorders Image taken from [12]

Childhood abuse is rising as a public health concern. Psychological impacts of childhood maltreatment extend in later adulthood, and vulnerability to common psychiatric conditions like depression and anxiety increases through to later age [11]. Traumatic experiences in childhood lead to the development of negative memory bias, which is strongly associated with comorbidities including a number of psychiatric disorders, as mentioned in Figure 1.

Several reviews have also explored the role of genetic polymorphism as a mediator of specific effects (suicidogenic, depressogenic and/or anxiogenic) of child abuse and neglect; these effects exert deleterious effects of early childhood adversity [13]. Childhood trauma also impairs the cortisol activating response (CAR) in adults with EDs and has a negative dose-dependent impact on HPA axis activity [14]. Additionally, there is a strong link between childhood maltreatment and negative affect. Research has shown that negative, depressive effects mark the severe psychopathology associated with poor outcomes in weight and eating problems [15]. This negative effect exerted by childhood maltreatment is believed to contribute to EDs by promoting feelings of shame, fears, guilt, body image problem, and diminished self-esteem [16-18].

Child Abuse and EDs

Childhood abuse is thought to be linked to EDs through the development of psychiatric disorders. Related general psychopathology is apparent in psychiatric disorders like depression, anxiety, obsessive-compulsive, and increased interpersonal sensitivity. This psychopathological interaction can lead to EDs. Interestingly, there has been a debate in the past that emotional neglect is more independently associated with EDs regardless of co-occurrence of other psychiatric disorders [19]. ED cover a broad range of subtypes corresponding to behavioral characteristics of certain symptoms [20]. Also, many characteristic symptoms of EDs are the result of a complex interaction between environmental and genetic factors [21]. A vital construct of EDs is the parental invalidation of a child’s emotional needs, which is associated with difficulty in tolerating stress in individuals with EDs [22]. AN is the most well-known of these EDs and is characterized by an obsession for extreme food restriction and weight control [20]. AN could happen as full disease or could be associated with BN, where AN is considered a negative prognostic indicator in BN [23]. There have been contradictory findings on the link between childhood abuse and symptoms related to body dissatisfaction and urge for thinness both of which are behavioral characteristic of AN [24-26]. However, several studies have shown a strong correlation between emotional child abuse and/or neglect and AN. Table 1 mentions the studies showing the significant link between emotional child maltreatment and AN.

**Table 1:** Studies showing positive co-relation between emotional child maltreatment leading to emotional dysregulation as a mediator in developing anorexia nervosa AN, anorexia nervosa; BN, bulimia nervosa; BED, binge eating disorder; ED, eating disorder; AOR, adjusted odds ratio; CEA, childhood emotional abuse; PTSD, post-traumatic stress disorder [7], [10], [19], [27-29]

	Author/year	country	Study’s focus	results	findings
1.	Copeland et al. (2015)	United States	The study explores the effects of bullying in children on eating disorders, including AN and BN.	Victims of bullying were more likely to be underweight than those who were not involved in bullying; 46.7% vs. 36.1%. p <0.001.	These associations could be explained in part by the well-established emotional sequelae of bullying involvement such as anxiety or depressive symptoms. This is particularly the case with victims and bully-victims who are at risk for elevated depression and anxiety.
2.	Racine et al. (2014)	United States	The author focuses light on the impact of all types of child abuse on patients with AN with special emphasis on the mediation through emotional dysregulation.	CEA was significantly associated with emotion dysregulation and AN symptoms; CEA-emotion dysregulation was significantly larger than childhood sexual abuse -emotion dysregulation relationship(Steiger's Z+2.00,p = 0.02), childhood physical abuse relationship with emotional dysregulation and AN was not significantly associated.	In sum, of the abuse experiences examined, CEA appears to be particularly crucial for emotion dysregulation in individuals with AN.
3.	Reyes-Rodriguez et al. (2011)	United States	The main objectives of this study were to describe the nature of traumatic events experienced and to explore the relation between PTSD and AN in a sample of women.	Of traumatic events experienced by AN patients, out of n = 33 RAN (restrictive type AN), n = 12.1 had domestic violence child, out of n = 40 PAN(purge type AN), n = 15 recalls domestic violence, child. The majority of participants with PTSD reported the first traumatic event before the onset of AN (64.1%, n = 66).	Association of PTSD with AN show strong co-relation of emotional dysregulation psychopathological link with long term comorbidity in patients with AN.
4.	Bradonecone (2008)	United States	This study examines differences between women with BN with and without a history of AN regarding eating pathology, personality, and childhood maltreatment.	Emotional neglect was reported in BNAN (p: 0.21), Emotional abuse in BNAN (P: 0.10) and physical abuse in BNAN (p: 0.001) as compared to patients with BN without AN.	Women with BN and history of AN had higher levels of dietary restraint and purging and lower body mass indices as well as higher levels of all forms of childhood neglect and abuse. Psychopathological link could be due to generalized anxiety disorder leading to eating disorders after childhood maltreatment.
5.	Afifi TO et al. (2017)	United States	The study explored the link between child abuse, including harsh physical punishment, physical abuse, sexual abuse, emotional abuse, emotional neglect, physical neglect, and exposure to intimate partner violence, and eating disorders in adulthood among men and women.	Study shows AOR in women with AN for emotional abuse in 2 groups (AOR1 and AOR2 after adjusting for confounding factors) as AOR1:2.08 (p <0.001) and AOR2:1.77 (p <0.01) as compared to irrelevant results in men with AN. For childhood exposure to IPV (intimate partner violence) in men with AN, AOR1 is 3.35 (p <0.001) and AOR2 is 2.95 (p-value <0.05) as compared to insignificant results in women with AN.	Among men, sexual abuse and physical neglect had the most robust relationship with eating disorders. Among women, sexual abuse and emotional abuse had the most robust relationships with eating disorders. Second, physical neglect and any family history of dysfunction were more strongly associated with BED compared to AN. As well, emotional neglect was more strongly associated with BN and BED compared to AN.
6.	Guillaume et al. (2016)	France	The study evaluates the independent relationships between the type of childhood trauma and the critical features of EDs in a sample of women who were diagnosed with AN, BN or BED.	Emotional abuse and emotional neglect were significantly associated with weight concern (p-value 0.001 and p-value 0.01), respectively.	Emotional abuse is most associated with EDs independent of comorbid psychiatric disorders.

Emotional Dysregulation as a Mediator between AN and Child Abuse

Emotional childhood maltreatment is one of the subtypes of child abuse. It includes emotional abuse, emotional neglect, exposure to IPV, and exposure to bullying; which could be a possible proxy of emotional abuse in the form of threats and mockery. Strong link has been observed between childhood abuse and EDs. Although, review in the past showed this link between physical and sexual abuse but data about emotional abuse is limited [29].

The impact of emotional childhood maltreatment through emotional dysregulation is observed in all types of AN including AN-restrictive type vs. binge-eating/purging type [7]. Emotional dysregulation in children who are punished for emotional expression leads to the development of emotional evasion, hesitation, and repression [30]. Another critical factor in developing a limited repertoire of emotional coping strategies is poor stress tolerance in abusive parents [31]. This emotional dysregulation leads to AN as the result of maladaptive coping mechanism to deal with stress. Childhood traumatic experiences leading to EDs through emotional dysregulation can also be explained by the related psychopathology of post-traumatic disorder (PTSD) and AN [28]. Hyperactivity of amygdala caused by a traumatic experience in childhood seems to play a role in emotional dysregulation, and abnormal fear conditioning [32-33].

Although not directly tested, it has also been observed in patients with AN. Miyake et al. reported hyperactive amygdala associated with the processing of negative words about body image in individuals with RAN, and BAN as compared to control individuals and those with BN [28,34]. Another aspect of emotional child maltreatment is bullying which happens at school and home or public places. It makes victims and bully-victims vulnerable to develop depression and anxiety, and the indirect pathway from bullying to EDs is through poor self-esteem and emotional symptoms. Interestingly, bully-victims were at increased risk of exhibiting anorexic symptoms, increased vomiting for weight control or overcoming with bodyweight/shape [27]. Emotional childhood maltreatments through emotional dysregulation can also predispose to AN after developing insecure attachment styles. Studies have found the increased presentation of insecure attachment style in adolescents with AN [35]. Although studies about sexual and physical abuse are numerous, no reviews explored the emotional neglect as the factor involved in BN pts with or without a history of AN. This is another weakness in the literature [10]. A recent meta-analysis showed that childhood abuse is related to BN and BED, but there are no significant conclusions mentioned about AN [36].

Emotional child abuse is strongly linked to the development of Eds symptoms independent of co-occurrence of psychiatric co-morbidities in a sample of patients with EDs [19]. The underlying mechanism could be the specific impact of this type of abuse on emotional regulation. This is in contrast to the association of morbid psychopathology of childhood emotional maltreatment with AN through generalized anxiety disorders [10]. This is comparable to a study conducted in females with h/o sexual and physical abuse in which the results highlighted the symptoms of depression, PTSD and suicide attempts as the co-morbid psychiatric conditions in groups of individuals with EDs symptoms [20]. Talking about the subtypes of childhood abuse , emotional maltreatment showed strong impact on EDs in AN but there has also been evident association between physical abuse and AN; however sexual abuse was not reported to be a significant factor in the development of restrictive symptoms in AN, although they were reported in AN-binge-eating/purging type [7,37]. This is in contrast to sexual abuse impact on the development of Eds in other studies [37-39].

Other Psychological Mediators between Childhood Emotional Abuse and AN

Other mediators involved in child emotional maltreatment are through anxiety disorders and depressive disorders. Maltreatment is associated with generalized anxiety disorders, thus exploring the fact that anxiety disorders and BN with a history of AN may be connected via association with maltreatment; this is a new focal point in BN patients. Moreover, the studies are limited as the majority of studies on BN with history of AN were performed in the 1980s and 1990s when certain assessment methods, currently considered as the best tools to assess their intended constructs were not yet advanced or frequently used [10].

Gender Differences in the Impact of Emotional Child Maltreatment

Marked gender differences exist on the effect of emotional child maltreatment and later outcomes in adulthood with EDs. One study explored the gender differences very clearly with emotional abuse being more common in women [29]. The possible explanation is that women with a history of childhood trauma and having the lower expression of the genotype of 5-HTTLPR genes are emotionally sensitive and recognize fear and anger expressions more quickly. This emotional sensitivity plays a vital role in developing psychiatric disorders, which have a prominent emotional component [40]. Consequently, females are more likely to develop dysregulated eating behaviors as the long-term sequelae of childhood emotional maltreatment.

Limitations of Literature

The main weakness of literature lies in the type of study design, which is mostly retrospective cross-sectional or observational with a high risk of potential confounding and recall bias. Longitudinal studies exploring the temporal relationship between childhood emotional maltreatment and EDs, especially AN, should be considered in the future. Thus, the high-risk patients having AN with a history of emotional childhood maltreatment can be treated and managed accordingly to decrease the long-term morbidity associated with these EDs. Overall, this article adds specificity to previous research on the relation of emotional child maltreatment with ED psychopathology and the mediation of this general relation by emotional dysregulation through negative affect, generalized anxiety, and major depressive disorders.

Role of Social Implementations and CBT in Targeting Emotional Dysregulation

From a public health policy perspective, it is essential to realize the strong relationship between childhood maltreatment and EDs. There is a need to explore the mechanisms of these relationships to guide through developing preventive measures and treatment strategies [41]. Also, it is necessary to consider gender differences in making public policies; including men also, according to the type of child maltreatment type and specific ED behaviors [29]. Further investigation of the association of this subtyping should test for different treatment outcomes and psychosocial quality of life. For example, BN with or without a history of AN could have a different response to treatments based on various underlying etiologies [10]. After exploring the strong psychopathological link between emotional child abuse and AN, it would be wise to use evolving and advanced versions of CBT for EDs [42]. It would help us develop adaptations of dialectical behavioral therapy for EDs to implement the targeted strategies for assisting patients in coping with emotional regulation, negative affect, distress tolerance and hence may be very useful in decreasing the long term morbidity [43]. For example, treating depressive symptoms and improving self-esteem may improve meaning in life in patients with severe mental disorders who were affected by CM [44].

Also, gender differences may signal the need for different assessment tools in EDs in males and females [15]. Social support is another strong aspect of the treatment options in patients with AN as it will target the emotional component of disordered eating behaviors, having long term consequences. There is a hope that the development of multi-leveled ED models can lead to making different testable hypotheses relevant to future psychological assessment and treatment of EDs [45].

## Conclusions

As an indispensable and critical public health concern, childhood maltreatment and EDs are associated with significant mortality and morbidity. The present review and critical systematic search emphasize on the psychopathological correlation of emotional childhood maltreatment with AN and raises significant concerns and questions about the extent and the results of the literature on emotional child abuse, emotional child neglect, and exposure to IPV. Based on our review, it is apparent that a significant portion of patients with AN have reported a history of exposure to emotional childhood maltreatment. Our critical review indicated that researchers have focused on the physical and sexual childhood maltreatment more than emotional maltreatment. So, the literature focusing on emotional childhood maltreatment is lacking and is the least type of child abuse being addressed in the clinical setting as well as the public health level. The limited data raises the concern about the need to conduct more studies to explore the temporal causal relationship between emotional childhood maltreatment and AN. Future research should also consider whether targeting emotional dysregulation will benefit patients with AN.
